# Ultrasonic-Assisted Efficient Extraction of Coumarins from *Peucedanum decursivum* (Miq.) Maxim Using Deep Eutectic Solvents Combined with an Enzyme Pretreatment

**DOI:** 10.3390/molecules27175715

**Published:** 2022-09-05

**Authors:** Zeyu Li, Qian Li

**Affiliations:** State Key Laboratory of Aridland Crop Science, College of Agronomy, Gansu Agricultural University, Lanzhou 730070, China

**Keywords:** *Peucedanum decursivum* (Miq.) Maxim, enzyme, deep eutectic solvents, coumarins, antioxidant activity

## Abstract

In this study, the ultrasonic-assisted extraction of total coumarins from *Peucedanum decursivum* (Miq.) Maxim (*P. decursivum*) via the combination of deep eutectic solvents (DESs) with cellulase pretreatment was carried out. Among the 15 kinds of DESs with choline chloride as hydrogen bond acceptors, the DES system of choline chloride/1,4-butanediol with a molar ratio of 1:4 showed the best extraction effect. First, single-factor experiments were performed using the following factors: liquid–solid ratio, pH, enzyme dosage and ultrasonic temperature. The Box–Behnken design (BBD) and response surface methodology (RSM) were employed to optimize the extraction conditions and obtain the following optimal parameter values for the extraction of coumarins from *P. decursivum*: liquid–solid ratio 14:1 mL/g, pH 5.0, enzyme dosage 0.2%, ultrasonic temperature 60 °C and ultrasonic time 50 min. Under these conditions, the extraction yield of total coumarins from *P. decursivum* could reach 2.65%, which was close to the predicted extraction yield of 2.68%. Furthermore, the contents of six coumarins, namely, umbelliferone, nodakenin, xanthotoxin, bergapten, imperatorin and decursin were determined to be 0.707 mg·g^−1^, 0.085 mg·g^−1^, 1.651 mg·g^−1^, 2.806 mg·g^−1^, 0.570 mg·g^−1^ and 0.449 mg·g^−1^, respectively, using HPLC-MS after the optimization. In addition, the cell fragmentation of *P. decursivum* powder obtained using ultrasonic-assisted DES extraction with enzyme pretreatment was found to be the most comprehensive using scanning electron microscopy (SEM), which indicated the highest extraction efficiency for *P. decursivum*. Finally, the in vitro antioxidant activity of the extracts was evaluated via radical scavenging with 1,1-diphenyl-2-picrylhydrazyl (DPPH), which showed that ultrasonic-assisted DES extraction with enzyme pretreatment exhibited significant antioxidant activity with DPPH radical scavenging of up to 97.90%. This work developed a new and efficient extraction method for coumarins.

## 1. Introduction

*Peucedanum decursivum* (Miq.) Maxim (*P. decursivum*) is the dry root belonging to Umbelliferae [1]. It has a very wide distribution throughout the world, not only in China but also in Japan, Korea, India, etc. [2]. *P. decursivum* was confirmed to possess a wide range of pharmacological activities, including expectorant and antiasthmatic, antioxidant, antibacterial, anti-inflammatory, anticoagulant, and anticancer properties [3]. Coumarins are one of the main chemical components in *P. decursivum*. In addition, there are flavonoids, volatile oils, saponins and other compounds in *P. decursivum* [4]. Coumarins were first reported in 1820, and so far, there are more than 1300 kinds of coumarins to be found [5]. *P. decursivum* mainly contains nodakenin, xanthotoxin, scopoletin, decursin and other coumarins [6]. Nodakenin is used as the detection index of coumarins in *P. decursivum*, as stipulated in the *Pharmacopoeia of the People’s Republic of China*. The efficient extraction of coumarins from *P. decursivum* is demanding.

Traditional methods for extracting coumarins include organic solvent immersion, ethanol reflux and CO_2_ extraction. These methods have the disadvantages of using a toxic extraction solvent, being time-consuming and needing high energy consumption. Ultrasonic waves enhance the extraction effect due to the acoustic cavitation effect (mechanical and chemical effect) produced by ultrasonic waves passing through the solvent. Ultrasonic-assisted extraction (UAE) has the advantages of a short required time and low solvent usage [7,8]. It is well known that plant cell walls are mainly composed of cellulose. Extraction with cellulase could destroy the plant cells, improve the permeability of the cell walls and facilitate the dissolution of effective components. The combination of enzymolysis with ultrasonic extraction could improve the permeability of cells and is more conducive to the dissolution of effective components [9,10]. This combination of the techniques was widely used in the extraction of traditional Chinese medicines [11,12].

The use of a deep eutectic solvent (DES) was first proposed by Abbott et al. in 2003 [13]. With the development of green chemistry, the application of DESs has attracted great attention due to their advantages of biodegradability and good biocompatibility [14]. In recent years, DESs have developed rapidly in analytical research, catalysis and organic synthesis [15,16,17]. In particular, DESs were used for the extraction and separation of bioactive compounds, such as phenolic acids, flavonoids and alkaloids from various plant materials with high extraction efficiencies [18,19]. The lower toxicity and higher biodegradability of DESs were mainly assigned to the group of DESs composed of natural, low-toxicity compounds [20]. The toxicities of DESs were evaluated by using prokaryotic microorganisms, which focused on Gram-positive and Gram-negative bacterial strains, yeast strains and mold fungi strains [21,22,23]. For example, the toxicity of choline chloride (ChCl)-based DESs in three bacterial strains (*E. coli, P. aeruginosa* and *S. aureus*) were evaluated qualitatively and quantitatively, and it was concluded that both ChCl:urea and ChCl:glucose seem to have low toxicity, and thus, may be referred to as “green solvents” [24]. The current studies on the microbial toxicity of DESs showed that they were generally less toxic to different microorganisms and were less toxic than traditional organic solvents; therefore, the use of DESs is encouraged [25].

DESs are composed of hydrogen bond acceptors (HBAs) and hydrogen bond donors (HBDs). The hydrogen bond acceptors are usually quaternary ammonium salts, alkaloids and amino acids. There are more kinds of hydrogen bond donors, including urea, polyols, organic acids and sugars. DESs gradually decompose with an increase in temperature, mainly because the hydrogen bond is a weak intermolecular force. The increase in temperature weakens the van der Waals force and hydrogen bond interaction in choline-based DESs [26], and the hydrogen bond would break to a certain extent. Chen et al. [27] studied 29 kinds of DESs with ChCl as an HBA, and the results showed that different DESs had different initial decomposition temperatures, where the lowest was ChCl:acetic acid (34.3 °C), and most DESs initial decomposition temperatures were above 100 °C. The decomposition process of DESs indicated that the HBDs had relatively poor thermal stability and would decompose first at their characteristic decomposition temperature, followed by ChCl, and the rest would decompose at about 250 °C to 300 °C. Most studies [28,29,30] showed that the temperature of the ultrasonic extraction method was lower than that of the traditional heating reflux method, which could reduce the decomposition of DESs, improve the thermal instability of DESs and avoid the degradation of thermally unstable compounds at higher temperature [31]. The various physical properties of DESs, as well as the large difference in the solubility of the active materials, were developed according to the different compositions and water contents. Therefore, it is necessary to select suitable DESs for extraction [32]. Ultrasonic-assisted extraction (UAE) coupled with DESs has developed as an efficient and green extraction method for natural products. The combination of UAE, DESs and enzymolysis for the extraction of phytochemicals is less developed, although this combination may provide higher extraction efficiency.

In this study, a series of DESs based on ChCl were developed. The ultrasonic-assisted extraction of total coumarins from *P. decursivum* using a combination of DESs with cellulase pretreatment was carried out. A Box–Behnken design (BBD) and response surface methodology (RSM) were employed to optimize the extraction conditions. Based on the optimal extraction process, the HPLC-MS method for the determination of six coumarins in *P. decursivum* was established for the first time. Scanning electron microscopy (SEM) was used to investigate the crushing degree of pharmaceutical powders via ultrasound-assisted DES and traditional extraction solvents. Finally, the DES extracts under optimal conditions were investigated for their antioxidant activity. 

## 2. Results and Discussion

### 2.1. Screening of Deep Eutectic Solvents

**Figure 1:**
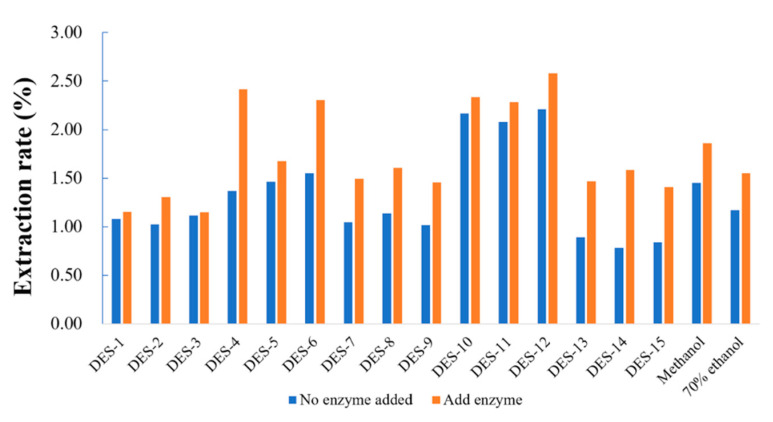
Extraction yield of total coumarins using different organic solvents and DESs.

In order to understand the advantages of DESs in extracting total coumarins with enzyme pretreatment from *P. decursivum*, the extraction yields of different organic solvents (methanol, 70% ethanol) and different types of DESs were compared; the results are shown in Figure 1. Under the conditions of ultrasonic power 300 W, extraction temperature 40 °C and extraction time 40 min, the deep eutectic system composed of choline chloride/1,4-butanediol (molar ratio 1:4) had a significantly higher extraction effect on the extraction of coumarin components of *P. decursivum* than the other DESs and traditional extraction solvents.

The physical properties of the DESs, such as hydrogen bonding force, polarity and viscosity, directly affect the extraction yield of target compounds and the hydrogen bonding effect is the main factor that affects the solubility of DESs [33]. Too low viscosity indicates that there are fewer hydrogen bonds between the components of the DESs and extraction of the target compound is not utilized. Too high viscosity can limit the mobility of target compounds within DESs and reduce the extraction yield, which can affect the extraction effect [34]. The addition of cellulase could promote the destruction of the plant cell walls and accelerate the efflux of contents. Eventually, a eutectic system composed of choline chloride/1,4-butanediol (1:4) was selected as the extraction solvent according to the extraction yield (2.58%) of total coumarins.

### 2.2. Single-Factor Experimental Analysis of Extraction of Coumarins from P. decursivum

#### 2.2.1. Effect of the Liquid–Solid Ratio

**Figure 2:**
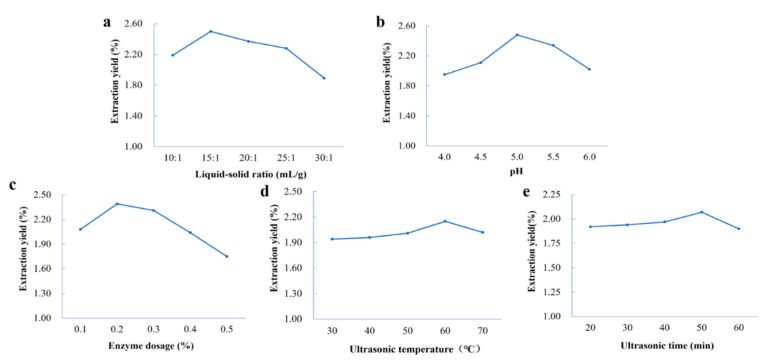
Single-factor effects on the extraction yield of total coumarins: (**a**) liquid–solid ratio, (**b**) pH, (**c**) enzyme dosage, (**d**) ultrasonic temperature and (**e**) ultrasonic time.

The effects of different liquid–solid ratios (10:1, 15:1, 20:1, 25:1, 30:1 mL/g) on the extraction yield of total coumarins were investigated under certain sonication (300 W, 50 Hz, 40 min, 40 °C), enzyme dosage (0.2%) and pH (5.0) conditions; the results are shown in Figure 2.

As shown in Figure 2a, the total coumarins yield increased first and then decreased with the increase in the liquid–solid ratio. The extraction yield reached a maximum at 2.50% when the liquid–solid ratio was 15:1. This may have been due to the increase in the liquid–solid ratio, which increased the contact area between the liquid and solid, caused the materials to fully infiltrate, promoted the diffusion of coumarins components to the extraction solvents and improved the extraction yield of total coumarins. However, when the liquid–solid ratios were too small, only a small number of samples were processed within a certain period and the solvent was not fully utilized. When the liquid–solid ratio was too large, the energy conduction of ultrasonication was impeded, resulting in a smaller volume of liquid subjected to ultrasonication, slower solvent dispersion and weakening of cell wall fragmentation; therefore, this was unfavorable for the dissolution of coumarin components, resulting in a lower total coumarins extraction yield [35].

#### 2.2.2. Effect of the pH

As shown in Figure 2b, with the increase in pH, the extraction yield of total couma-rins first increased gradually. After the pH exceeded 5.0, the extraction yield began to de-crease. This may have been due to the fixed optimal pH value of enzymes, and overacidity or overbaseline would affect the activity of enzymes. The reduction of cellulase activity would directly affect the effect on plant cell walls, thus affecting the extraction yield [36]. Therefore, the enzymatic solution system pH was chosen to be appropriate at 5.0.

#### 2.2.3. Effect of the Enzyme Dosage

As shown in Figure 2c, when the dosage of cellulase was 0.2%, the extraction yield of total coumarins was the highest. When the dosage of cellulase continued to increase, the extraction yield of total coumarin gradually decreased. With the increase in enzyme dosage, the target compound was fully combined with the enzyme to promote the continuous dissolution of substances. When the dosage of cellulase exceeded 0.3%, the enzymes were too saturated relative to the target compound, which was not conducive to the overflow of coumarins [37]. Therefore, the best proportion of enzyme dosage was 0.2%.

#### 2.2.4. Effect of Ultrasonic Temperature

As shown in Figure 2d, the temperature was positively correlated with the total coumarins extraction yield between 30 °C and 60 °C. The higher temperature could reduce the viscosity of DESs and increase the mass transfer effect [38], thus increasing the total coumarins extraction yield. From 60 °C to 70 °C, the extraction yield showed a decreasing trend, which indicated that too high a temperature might cause the target product to be destroyed and increase the dissolution of impurity components. On the other hand, too high a temperature could cause enzyme inactivation, affecting the total coumarins extraction yield. Therefore, 60 °C was a suitable sonication temperature for total coumarins extraction from *P. decursivum*.

#### 2.2.5. Effect of Ultrasonic Time

As shown in Figure 2e, the maximum extraction yield of 2.07% was obtained at 50 min. After 50 min, the total coumarins extraction began to decrease with the extension of time. The results indicated that within a certain period, the cavitation effect of the ultrasonic waves was complete, the interaction between sample and solvent was sufficient [39] and the extraction yield did not change significantly with the increase in ultrasonic time. Too long an ultrasonic time would lead to the continuous dissolution of other components in *P. decursivum*, increase the viscosity of the extract and reduce the dissolution of the target substance. Therefore, the ultrasonic extraction time should be 50 min.

### 2.3. Optimization of the Extraction Conditions Using a BBD RSM

In the above experiments, the choline chloride/1,4-butanediol (1:4) system combined with enzyme pretreatment was demonstrated to be the best extraction method. The single factors that had the largest effects on *P. decursivum* were selected as variables (liquid–solid ratio, pH and enzyme dosage), the total coumarins extraction yield (Y) was taken as the response value, and the Box–Behnken (BBD) design was performed by using the software Design-Expert v8.0.6.1 to generate the three-factor and three-level response surface. The response test factors and levels are shown in Table 1.

In this study, the extraction conditions were optimized and a total of 17 experiments were performed (Table 2).

The software Design-Expert v8.0.6.1 was applied to analyze the data and obtain optimal results using different parameters. Multiple regression analysis of the experimental data yielded a regression equation for the total coumarins extraction yield, which was fitted as a quadratic polynomial as follows:

Y = 2.66 − 0.12 × A − 0.0075 × B + 0.10 × C + 0.058 × AB + 0.025 × AC − 0.13 × BC − 0.44 × A^2^ − 0.56 × B^2^ − 0.41 × C^2^ (1)

where Y is the predicted extraction yield of total coumarins from *P. decursivum*, and A, B and C are the liquid–solid ratio, pH and enzyme dosage parameters, respectively.

Regression models were applied for statistical significance, and the analysis of variance results for the response surface data is shown in Table 3.

As can be seen from Table 3, the regression equation had *p* < 0.01, indicating that the regression model reached a highly significant level. The coefficient of determination of the model reached 0.9876, which indicated that the model could explain 98.76% of the variation of the response value. The lack-of-fit term was not significant, indicating that the equation fit the test well and that the test error was small. The F-value of the model was 61.80, indicating that the model building was effective. The effects of the three factors A, B and C on the extraction yield of coumarins from *P. decursivum* were judged according to the magnitude of the F-value, and the order of their magnitudes was as follows: A (liquid–solid ratio) > C (enzyme dosage) > B (pH).

### 2.4. Response Surface Analysis Diagram

**Figure 3:**
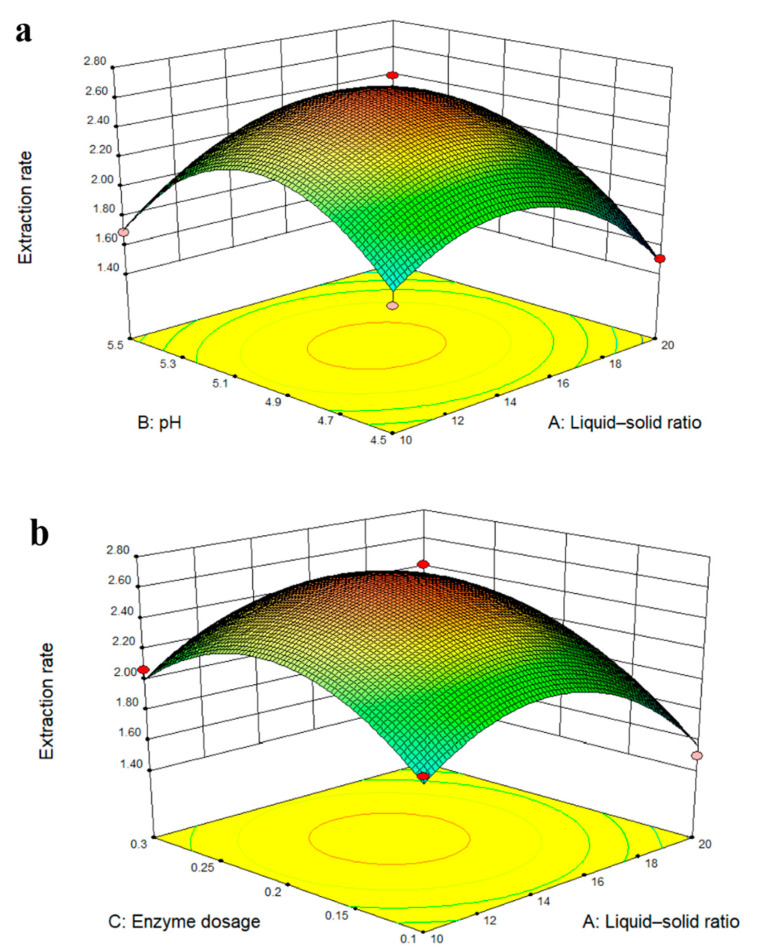

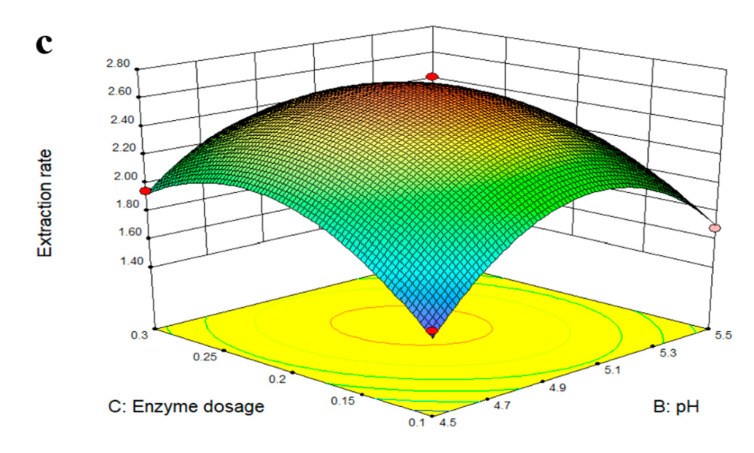
Response surface analysis diagram: (**a**) the reciprocal response of the liquid–solid ratio and pH, (**b**) the reciprocal response of the pH and enzyme dosage, and (**c**) the reciprocal response of the liquid–solid ratio and enzyme dosage.

The steepness of the response surface reflects the significance of the interaction between the two factors, where the steeper the surface, the higher the significance. The interaction effects between the factors are shown in Figure 3.

The response surface was a smooth surface that opened downward, indicating that the maximum extraction yield of total coumarins of *P. decursivum* could be obtained within the designed horizontal range [40]. The curve for pH and enzyme dosage was the steepest, which indicated that the interaction between pH and enzyme dosage had the most significant effect on the response value. This was consistent with the analysis of variance results shown in Table 3.

### 2.5. Validation of the Prediction Model

Validation tests were performed for the extraction process of total coumarins after the optimization of combined tests in the Box–Behnken center with three replicates. Using this regression equation, the optimal extraction conditions were determined to be a liquid–solid ratio (A) of 14.37:1, a pH (B) of 4.98 and an enzyme dosage (C) of 0.21%, and the model predicted a maximum extraction yield of 2.68%. Considering the practical operability, the optimal conditions were adjusted to be liquid–solid ratio 14:1, pH 5.0 and enzyme dosage 0.2%, and the total coumarins extraction yield was 2.65% from three parallel validation experiments under these conditions, which indicated that the experimental results were in good accordance with the model and the regression equations.

### 2.6. Contents Analysis of Six Coumarins of P. decursivum

On the basis of the above experimental optimization conditions, HPLC-MS was applied to determine the contents of the six coumarin compounds in *P. decursivum*. The contents of umbelliferone, nodakenin, xanthotoxin, bergapten, imperatorin and decursin in *P. decursivum* were determined to be 0.707 mg·g^−1^, 0.085 mg·g^−1^, 1.651 mg·g^−1^, 2.806 mg·g^−1^, 0.570 mg·g^−1^ and 0.449 mg·g^−1^, respectively.

### 2.7. Microstructure of the Plant Powder

**Figure 4:**
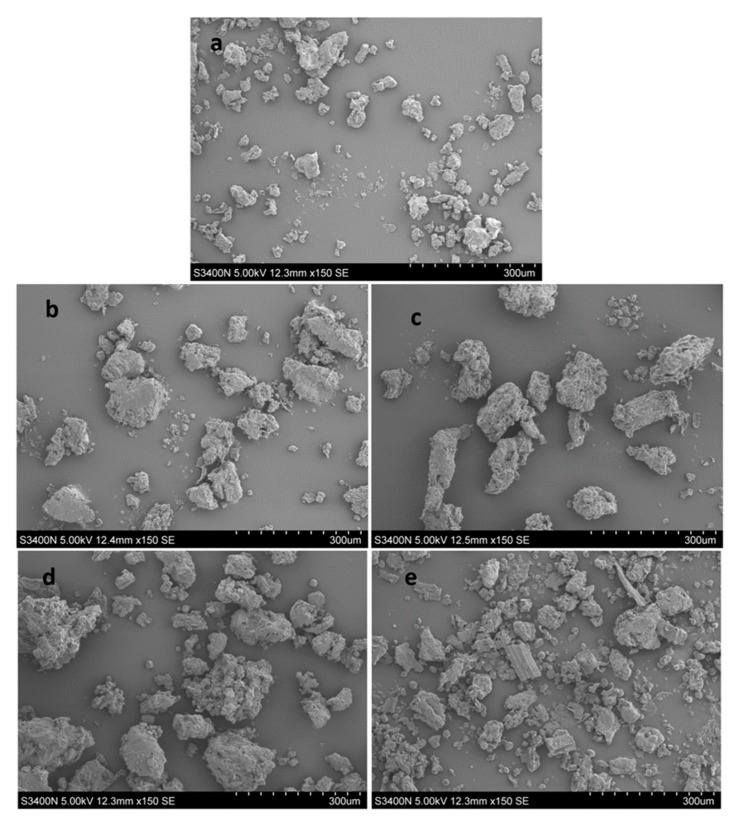
The SEM photos of (**a**) the unextracted crude drug powder, (**b**) ultrasonic-assisted methanol extraction with enzyme pretreatment, (**c**) ultrasonic-assisted 70% ethanol extraction with enzyme pretreatment, (**d**) ultrasonic-assisted DESs extraction and (**e**) ultrasonic-assisted DES extraction with enzyme pretreatment.

The powder treated using different extraction methods was observed using scanning electron microscopy (SEM). As shown in Figure 4, the powdered cells obtained using DES with an enzyme pretreatment were the most severely broken, which indicated that cellulase could more effectively destroy the tissue structure of *P. decursivum* by hydrolyzing cellulose and hemicellulose on the cell walls to improve the extraction yield. Regarding the enzyme-assisted methanol extraction and enzyme-assisted 70% ethanol extraction, there were obvious cracks on the outer surface of the sample powder after the ultrasonic treatment, and loose and broken structures could be observed. Therefore, compared with the traditional extraction solvents, the ultrasonic extraction of coumarins from *P. decursivum* using DES with enzyme pretreatment was found to be an efficient and green extraction method.

### 2.8. Antioxidant Capacity of the Extracts

Modern scientific research points out that the production of excessive free radicals is often closely related to many chronic diseases and aging. Antioxidation can effectively reduce the harm it brings [41]. Studies showed that many coumarins have strong antioxidant effects, which could act as natural plant antioxidants [42]. Natural plant antioxidants demonstrate good antioxidant effects and have the advantages of small toxic side effects, high safety and a high content of active ingredients, which can delay human aging and enhance their own resistance.

**Figure 5:**
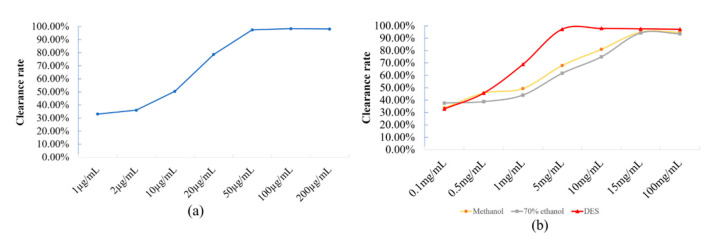
The DPPH radical scavenging activities of (**a**) the VC reference solution and (**b**) methanol, 70% ethanol and the DES solution.

DPPH has been widely used to determine the antioxidant capacity of biological samples, pure compounds and extracts in vitro. The method is simple and can be used to evaluate the antioxidant activity of samples [43,44]. In this study, the antioxidant activities of the enzyme-assisted extracts of three different extraction solvents (methanol, 70% ethanol and DESs) were compared. With VC as the control, the DPPH radical scavenging activities of three different treatment solutions were found and are shown in Figure 5.

As shown in Figure 5, with the increase in the concentration of different sample solutions, the scavenging ability of antioxidants in different solutions to DPPH free radicals gradually increased, reaching a certain concentration and then gradually tending toward being stable. The clearance rate of VC to DPPH tended to be stable when the concentration was 50 μg/mL, and reached the maximum at 100 μg/mL, with a maximum clearance rate of 98.41%. The DPPH clearance rate of the enzyme-assisted DES extract tended to be stable at 5 mg/mL, and the maximum clearance rate was 97.30%. When the enzyme-assisted methanol extract and 70% ethanol extract reached the specified concentration, their clearance rates were 95.00% and 94.30%, respectively, which were lower than those of the DES extract.

According to an SPSS 26.0 software analysis, the IC_50_ values extracted using enzyme-assisted methanol, enzyme-assisted 70% ethanol and enzyme-assisted DES are shown in Table 4. The smaller the IC_50_ value, the stronger the antioxidant capacity. In conclusion, the VC control solution had the strongest DPPH radical scavenging ability. The scavenging ability of the enzyme-assisted DES extract was better than that of enzyme-assisted methanol and the enzyme-assisted 70% ethanol extract.

## 3. Materials and Methods

### 3.1. Plant Materials

*Peucedanum decursivum* (GAUAB-PD-20211010) was purchased from Dejiang County (Guizhou, China) and was identified by Professor Yuan Chen, Department of Chinese Herbal Medicine of Gansu Agricultural University (Lanzhou, China). The medicinal materials were put into an oven and baked at 60 °C for 24 h; they were then crushed with a 40 mesh sieve to obtain the coarse powder of *P. decursivum*.

### 3.2. Chemicals and Reagents

Nodakenin (lot: 20070604, purity ≥ 98%), umbelliferone (lot: 20052901, purity ≥ 98%), bergapten (lot: 17120501, purity ≥ 98%), xanthotoxin (lot: 17111503, purity ≥ 98%), imperatorin (lot: 18051502, purity ≥ 98%) and decursin (lot: Z08O9X71726, purity ≥ 98%) were purchased from Chengdu Pfeidian Biotechnology Co., Ltd. (Chengdu, China). Choline chloride (lot: C13957234, purity = 98%), 1,3-butanediol (lot: C12854459, purity = 99%), 1.4-butanediol (lot: C13190733, purity = 98%) and urea (lot: C13811737, purity = 99%) were purchased from Shanghai Macklin Biochemical Technology Co., Ltd. (Shanghai, China). Lactic acid (lot: 202101002, purity ≥ 90%), glycerol (lot: 20220102, purity ≥ 99%), methanol (lot: 20210608, purity ≥ 99.9%) and anhydrous ethanol (lot: 20211102, purity ≥ 99.7%) were purchased from Tianjin Fangzheng Reagent. All the above reagents, except methanol, were analytical reagents. In addition, there are 1,1-Diphenyl-2-picrylhydrazyl (DPPH) (lot: UFQ2I-WL, purity > 97%), cellulase (lot: CC31152810) and deionized water.

### 3.3. Establishment of a Method for the Determination of Total Coumarins in P. decursivum Using UV-VIS Spectroscopy

#### 3.3.1. Preparation of the Reference Solution

A total of 10 mg of nodakenin standard was accurately weighed and put into a 10 mL volumetric flask, the methanol was added to dissolve and dilute, and the flask was closed and shaken well. A reference solution with a concentration of 1 mg/mL was prepared and put into a 4 °C refrigerator for future use.

#### 3.3.2. UV-VIS Spectroscopy Conditions

The UV-VIS spectral scanning at 200~400 nm of the nodakenin reference solution and sample solution was conducted. The absorption peak at 335 nm was selected for the quantitative analysis.

#### 3.3.3. Drawing the Standard Curve

A series of concentrations of the reference solution was obtained by diluting the reference solution with methanol and the absorbance was measured at 335 nm. The standard curve was drawn with the absorbance as the ordinate (y) and the concentration of the reference solution (μg/mL) as the abscissa (x). The curve equation was as follows: y = 0.0398x + 0.2399, r^2^ = 0.9985. This showed that nodakenin has good linearity in the concentration range of 4~20 μg/mL.

### 3.4. Total Coumarins from P. decursivum Using Enzyme-Assisted DES Extraction

#### 3.4.1. Preparation of the DESs

A certain molar ratio of HBA and HBD was put in a 250 mL conical flask, which was heated and stirred in a constant-temperature water bath (80 °C) until a stable, uniform, colorless and transparent liquid formed [45,46,47,48,49]. The prepared DESs of different systems are shown in Table 5.

#### 3.4.2. Enzyme Pretreatment

The experiment was carried out by referring to Liu et al.’s methods [50], though slight modifications were made. The *P. decursivum* powder (2.0 g) was put into a 250 mL conical flask with a stopper, 20 mL of DES solution with a water content of 30% was added according to the liquid–solid ratio of 20:1, and the pH 5.0 was adjusted with 0.3% sulfuric acid solution and 0.1 mol·L^−1^ sodium hydroxide solution. Then, 0.2% cellulase was added, which was stirred and reacted at a constant temperature (45 °C) for a certain time (magnetic stirring enzymolysis 800 r/min). Finally, the reaction unit was put into a 75 °C constant-temperature water bath for 10 min to kill the enzyme and it was cooled to room temperature.

#### 3.4.3. Ultrasonic Extraction

Ultrasonic treatment was carried out on the above enzymatic hydrolysis mixture and the ultrasonic conditions were set as follows: power 300 W, frequency 50 Hz, temperature 40 °C and time 40 min; the mixture was then centrifuged at 8000 RPM r/min for 15 min. A total of 500 μL of the preparation solution was added to a 50 mL volumetric flask, the volume was fixed with methanol to the scale line, the absorbance was measured according to the determination method under the standard curve and each test was repeated three times. The extraction yield of total coumarins was calculated according to the following formula [51]:

(2) $$Y(\%)=\frac{C\times V\times N}{M}\times 100\%$$

where *N* (*N* = 100) is the dilution multiple, *C* is the total coumarins concentration of *P. decursivum* in the DES test solution (mg/mL), *M* is the mass of the *P. decursivum* powder (mg) and *V* is the volume of constant volume solution (mL).

### 3.5. Validation of the Method for the Determination of Total Coumarins in P. decursivum Using UV-VIS Spectroscopy

The standard solution was prepared using the method described in “Section 3.3.1” by repeating the measurement at 335 nm 6 times, the absorbance value was recorded and the RSD value was calculated as 1.04%, indicating that the instrument had good precision.

The test solution was prepared using the method described in “Section 3.4”; the absorbance value was measured at 335 nm at 0, 2, 4, 6 and 8 h, respectively; and the RSD value was calculated to be 1.30%, indicating that the test solution prepared in the experiment was stable within 8 h.

According to the method described in “Section 3.4”, six test sample solutions were prepared in parallel and their absorbance values were measured at 335 nm. The RSD of the six test sample solutions was calculated to be 1.59%, indicating that the reproducibility of this method was good.

The LOD and the LOQ were calculated on the basis of signal-to-noise ratios of 3.3 and 10, respectively. An appropriate amount of standard solution was prepared using the method described in “Section 3.3.1”, it was diluted by multiple ratios and was measured 6 times continuously at 335 nm. The absorbance value was recorded. The LOD and LOQ were 0.2360 μg·mL^−1^ and 0.7453 μg·mL^−1^, respectively.

About 0.5 g of the known content of *P. decursivum* sample was accurately weighed, and six samples were prepared in parallel. A certain amount of standard solution was added, and the test solution was prepared according to the method described in “Section 3.4”. The test was carried out at 335 nm. The calculated recovery rate was 98.75%. and the RSD value was 1.16%.

### 3.6. Single-Factor Experimental Design

The extraction parameters were optimized with the total coumarins extraction yield of *P. decursivum* as the index of the single-factor experiment.

The effect of the liquid-solid ratio on the extraction yield was studied under the conditions of pH 5.0, enzyme dosage 0.2%, ultrasonic power 300 W, frequency 50 Hz, ultrasonic temperature 40 °C and ultrasonic time 40 min. Different liquid–solid ratios of 10:1, 15:1, 20:1, 25:1 and 30:1 mL/g were selected for extraction, and the extraction yield was calculated.

The effect of pH on the extraction yield was studied under the conditions of liquid–solid ratio 20:1 mL/g, enzyme dosage 0.2%, ultrasonic power 300 W, frequency 50 Hz, ultrasonic temperature 40 °C and ultrasonic time 40 min. pH levels of 4.0, 4.5, 5.0, 5.5 and 6.0 were selected to extract *P. decursivum*, and the extraction yield was calculated.

The effect of enzyme dosage on the extraction yield was studied under the conditions of liquid–solid ratio 20:1 mL/g, pH 5.0, ultrasonic power 300 W, frequency 50 Hz, ultrasonic temperature 40 °C and ultrasonic time 40 min. The enzyme dosages of 0.1%, 0.2%, 0.3%, 0.4%, 0.5% were selected to extract the medicinal materials of *P. decursivum*, and the extraction yield was calculated.

The effect of ultrasonic temperature on the extraction yield was studied under the conditions of a liquid–solid ratio of 20:1 mL/g, pH 5.0, enzyme dosage 0.2%, ultrasonic power 300 W, frequency 50 Hz and ultrasonic time 40 min. The ultrasonic temperatures of 30 °C, 40 °C, 50 °C, 60 °C and 70 °C were selected to extract the medicinal materials of *P. decursivum*, and the extraction yield was calculated.

The effect of ultrasonic time on extraction yield was studied under the conditions of liquid–solid ratio 20:1 mL/g, pH 5.0, enzyme dosage 0.2%, ultrasonic power 300 W, frequency 50 Hz and ultrasonic temperature 40 °C. The ultrasonic times of 20 min, 30 min, 40 min, 50 min and 60 min were selected to extract *P. decursivum*, and the extraction yield was calculated.

### 3.7. Determination of Six Coumarins in P. decursivum Using HPLC-MS

#### 3.7.1. Chromatographic and Mass Spectrometric Conditions

The HPLC-MS experiments were performed on an Agilent 1290-6460 triple quadrupole mass spectrometer (Agilent Technologies Co. Ltd., Santa Clara, CA, USA). Chromatographic column: Agilent Eclipse Plus-C18 (150 mm × 2.1 mm, 1.8 micron). Mobile phase: 0.1% formic acid (A)—methanol (B), gradient elution (0~0.5 min: 5%~15% B, 0.5~0.8 min: 15%~28% B, 0.8~1.0 min: 28%~32% B, 1.0~1.2 min: 32%~38% B, 1.2~1.5 min: 38%~45% B, 1.5~4.5 min: 45%~65% B, 4.5~5.5 min: 65%~95% B, 5.5~6.0 min: 95%~5% B, 6.0~8.0 min: 95%~5% B). Column temperature: 35 °C, flow rate: 0.3 mL · min ^−1^ and injection volume: 10.0 μL.

Mass spectrometry conditions: the dry gas and atomizer was nitrogen, the temperature of the dry gas was 295 °C, the flow rate of the dry gas was 11 L·min ^−1^ and the atomizer pressure was 15 psi. For the electric spray ion source (ESI), the spray voltage was −4.0 kV and the positive monitoring mode and the multiple reaction scanning mode (MRM) were selected.

#### 3.7.2. Preparation of the Reference Solution

Proper amounts of umbelliferone, nodakenin, xanthotoxin, bergapten, imperatorin and decursin reference materials were accurately weighed, and the volume was fixed to 10 mL with methanol to prepare 1 mg·mL^−1^ reference solutions. Each of the above reference solutions was accurately removed, diluted with methanol to prepare a mixed reference solution with a series of concentrations and stored in a refrigerator at 4 °C for standby.

#### 3.7.3. Preparation of the Test Solution

The *P. decursivum* powder (2.0 g) was placed in a 100 mL conical flask, and the sample was extracted according to the optimized conditions discussed above. The test solution was filtered by using a 0.22 μm microporous membrane.

#### 3.7.4. Investigation of the Linear Relationship

**Figure 6:**
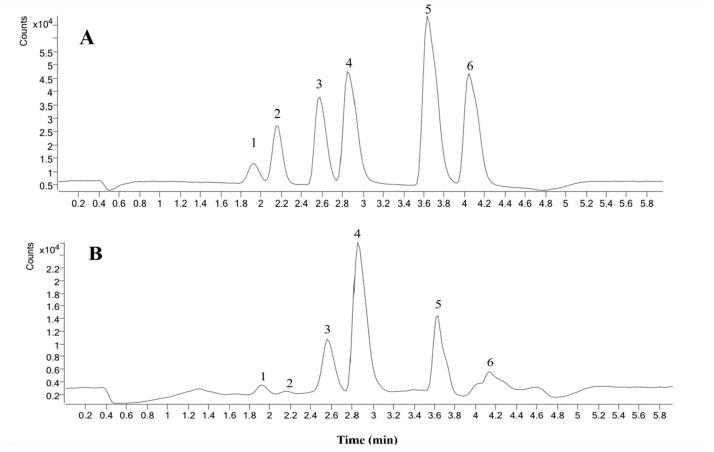
HPLC-MS chromatogram: (**A**) mixed standard solution and (**B**) sample solution. Note: 1 is umbelliferone, 2 is nodakenin, 3 is xanthotoxin, 4 is bergapten, 5 is imperatorin and 6 is decursin.

The 10 μL mixed reference solutions of different concentrations were injected into HPLC-MS device for analysis. The peak area (y) of each substance to be tested was the ordinate and the concentration (x) of each substance to be tested was the abscissa. Linear regression was carried out, where the regression equations of 6 components were calculated and the methodology was verified. The results are shown in Figure 6.

#### 3.7.5. Methodological Validation

The quantitative analysis of 6 coumarins in *P. decursivum* was verified using method validation. The linearity, precision, repeatability and stability were verified (Table 6). The calibration curves of 6 coumarins were performed with 6 standard solutions of different concentrations in triplicate. All calibration curves were highly linear with high correlation coefficients (R^2^ > 0.9941) over the test range. The results showed that the established method was accurate and sensitive for the quantitative analysis of 6 components of *P. decursivum*.

The mixture solution of the *P. decursivum* standard was injected 6 times in a row according to the chromatographic conditions described in “Section 3.7.1”. The peak area values were recorded and the RSD values were calculated. The RSDs of umbelliferone, nodakenin, xanthotoxin, bergapten, imperatorin and decursin were 0.68%, 1.19%, 0.45%, 1.02%, 0.68% and 0.60%, respectively, which indicated good precision of the instrument.

The samples were subjected to the chromatographic conditions described in “Section 3.7.1”. The RSDs of umbelliferone, nodakenin, xanthotoxin, bergapten, imperatorin and decursin were 0.71%, 0.97%, 0.59%, 1.04%, 0.89% and 1.41%, respectively. Therefore, the method had good repeatability.

Then, the prepared sample solution was analyzed under the chromatographic conditions described in “Section 3.7.3” at 2, 4, 8, 12 and 24 h. The peak area RSDs of umbelliferone, nodakenin, xanthotoxin, bergapten, imperatorin and decursin were 0.60%, 0.79%, 1.04%, 1.22%, 0.84% and 1.24%, respectively. Therefore, the results indicated that the test solution had good stability within 24 h.

An appropriate amount of mixed control solution was added to 1.0 g of the known content of *P. decursivum* following the treatment described in “Section 3.7.3” to prepare the test solution and the loading recovery was calculated. The RSDs were determined to be 1.49%, 1.40%, 0.68%, 1.56%, 1.38% and 2.04% for umbelliferone, nodakenin, xanthotoxin, bergapten, imperatorin and decursin, respectively.

### 3.8. Microstructure of the Plant Powders

The powders of *P. decursivum* treated using different extraction processes of the DES ultrasonic method, enzyme-assisted DESs extraction method, enzyme-assisted methanol extraction method and enzyme-assisted 70% ethanol extraction method were dried at low temperature and the microstructures were observed using scanning electron microscopy (SEM). In our experiment, the instrument model was a Hitachi S-3400N Scanning Electron Microscope. 

### 3.9. Determination of the Antioxidant Activity of Plant Extracts

In this study, DPPH free radical scavenging method was used to determine the antioxidant capacity. The antioxidant activities of extracts from three different extraction solvents (methanol, 70% ethanol and DES) assisted by enzymes were compared. Reference method [52]: With VC as the control, the three sample solutions were diluted according to different concentrations; then, 2 mL of sample solution with different concentrations and 2 mL of DPPH solution (0.1 mmol/L) were taken, which were added to 10 mL test tubes, shaken evenly, kept away from light at room temperature for 30 min, and the absorbance value was measured at 517 nm, which was recorded as A_x_. At the same time, the absorbance of DPPH was measured, which was recorded as A_0_, and the absorbance of 2 mL sample solution + 2 mL methanol was measured and recorded as A_1_. Using clearance = [1 − (A_x_ − A_1_) /A_0_] × 100%, the DPPH radical scavenging rates of different sample solutions were calculated, and the IC_50_ of each sample solution was calculated with SPSS 26.0. Each group of tests was performed three times.

## 4. Conclusions

In this study, coumarins were extracted from *P. decursivum* via ultrasonic extraction assisted by DESs combined with an enzyme pretreatment. First, different DES systems were prepared to extract the samples of *P. decursivum*. By comparing the extraction yields of different systems, it was found that the DES system composed of choline chloride and 1,4-butanediol with a molar ratio of 1:4 had the best effect when extracting coumarin from *P. decursivum*. Then, through the response surface optimization experiment design, the following optimal extraction conditions were obtained: liquid-solid ratio 14:1 mL/g, pH 5.0, enzyme dosage 0.2%, ultrasonic temperature 60 °C and ultrasonic time 50 min. Under this condition, the extraction yield can reach 2.65%, which is close to the theoretically predicted value, and thus, showed that the model was reasonable and reliable and could be used to optimize the process of extracting coumarins from *P. decursivum*. The content of six coumarin compounds, namely, umbelliferone, nodakenin, xanthotoxin, bergapten, imperatorin and decursin, in *P. decursivum* was determined using HPLC-MS. 

In addition, the results of the SEM showed that the cells of the medicinal material of *P. decursivum* treated using ultrasonic-assisted DES were the most seriously broken. Finally, the antioxidant activity of the extract in vitro was studied using a DPPH radical scavenging method. The results showed that the antioxidant activity of the DES extract was higher than that of the traditional solvent extract, and their IC_50_ values were 21.046 mg/mL, 24.088 mg/mL, and 25.512 mg/mL for extracts using enzyme-assisted DES, enzyme-assisted methanol and enzyme-assisted 70% ethanol, respectively. The application of this medicine in antioxidation could be further studied.

In summary, the comprehensive experimental results showed that the ultrasonic-assisted extraction method with DESs with high stability and low toxicity was more advantageous, which provided a theoretical basis and research basis for the comprehensive utilization of the resources of *P. decursivum*, the development of high value-added products and industrial transformation.

## Figures and Tables

**Table 1:** Response surface test factors and levels.

Single Factor	Level		
	−1	0	1
A (Liquid–solid ratio, mg/mL)	10	15	20
B (pH)	4.5	5.0	5.5
C (Enzyme dosage, %)	0.1%	0.2%	0.3%

**Table 2:** Box–Behnken test design scheme and results.

No.	A (Liquid–Solid Ratio, mg/mL)	B (pH)	C (Enzyme Dosage, %)	Y (Extraction Yield, %)
1	1	0	−1	1.50
2	0	0	0	2.65
3	−1	0	−1	1.89
4	0	0	0	2.63
5	0	−1	−1	1.51
6	1	0	1	1.78
7	1	−1	0	1.51
8	0	−1	1	1.95
9	1	1	0	1.68
10	0	1	−1	1.68
11	0	0	0	2.75
12	−1	−1	0	1.75
13	0	1	1	1.61
14	−1	1	0	1.69
15	0	0	0	2.63
16	0	0	0	2.66
17	−1	0	1	2.07

**Table 3:** ANOVA analysis of the Box–Behnken test.

Variables	Sum of Squares	df	Mean Square	F-Value	*p*-Value	
Model	3.48	9	0.39	61.80	<0.001	Significant
A: liquid–solid ratio	0.11	1	0.11	17.26	0.0043	
B: pH	0.00045	1	0.00045	0.072	0.7964	
C: enzyme dosage	0.086	1	0.086	13.75	0.0076	
AB	0.013	1	0.013	2.11	0.1895	
AC	0.0025	1	0.0025	0.40	0.5476	
BC	0.065	1	0.065	10.38	0.0146	
A^2^	0.82	1	0.82	131.33	<0.001	
B^2^	1.34	1	1.34	214.21	<0.001	
C^2^	0.71	1	0.71	114.11	<0.001	
Residual	0.044	7	0.006264			
Lack of fit	0.034	3	0.011	4.56	0.0884	Not significant
Pure error	0.00992	4	0.00248			
Cor total	3.53	16				
R^2^	0.9876					
Adj R^2^	0.9716					

**Table 4:** IC_50_ of the different extraction methods.

Extraction Method	IC_50_ (mg/mL)
Enzyme-assisted methanol	24.088
Enzyme-assisted 70% ethanol	25.512
Enzyme-assisted DESs	21.046

**Table 5:** List of DESs synthesized in this study.

No.	Hydrogen Bond Acceptors (HBAs)	Hydrogen Bond Donors (HBDs)	HBA/HBD Ratio	Appearance at Room Temperature
1	Choline chloride	Lactic acid	1:2	Light-yellow transparent liquid
2	Choline chloride	Lactic acid	1:3	Light-yellow transparent liquid
3	Choline chloride	Lactic acid	1:4	Light-yellow transparent liquid
4	Choline chloride	Glycerol	1:2	Transparent liquid
5	Choline chloride	Glycerol	1:3	Transparent liquid
6	Choline chloride	Glycerol	1:4	Transparent liquid
7	Choline chloride	1,3-Butanediol	1:2	Transparent liquid
8	Choline chloride	1,3-Butanediol	1:3	Transparent liquid
9	Choline chloride	1,3-Butanediol	1:4	Transparent liquid
10	Choline chloride	1,4-Butanediol	1:2	Transparent liquid
11	Choline chloride	1,4-Butanediol	1:3	Transparent liquid
12	Choline chloride	1,4-Butanediol	1:4	Transparent liquid
13	Choline chloride	Urea	1:2	Transparent liquid
14	Choline chloride	Urea	1:3	Transparent liquid
15	Choline chloride	Urea	1:4	Transparent liquid

**Table 6:** Validation of linear regression equation, linear range, precision, repeatability and stability of 6 coumarins.

Analyte	Calibration Curve	R^2^	Linearity Range (μg/mL)	Precision (RSD%)	Repeatability (RSD%)	Stability (RSD%)	Sample Recovery (RSD%)
Umbelliferone	y = 4.690579x − 306.776623	0.9947	0.16–6	0.68	0.71	0.60	1.49
Nodakenin	y = 15.913140x − 506.921696	0.9996	0.16–6	1.19	0.97	0.79	1.40
Xanthotoxin	y = 23.414009x − 2979.434097	0.9969	0.16–6	0.45	0.59	1.04	0.68
Bergapten	y = 40.140902x − 2041.418550	0.9966	0.16–6	1.02	1.04	1.22	1.56
Imperatorin	y = 75.809103x − 3549.463841	0.9986	0.16–6	0.68	0.89	0.84	1.38
Decursin	y = 46.962356x − 6888.543659	0.9941	0.16–6	0.60	1.41	1.24	2.04

## Data Availability

The data presented in this study are available on request from the corresponding author.

## Sample Availability

Samples of the plant materials of *Peucedanum decursivum* (Miq.) Maxim are available from the authors.
